# Adaptive Disturbance Rejection Motion Control of Direct-Drive Systems with Adjustable Damping Ratio Based on Zeta-Backstepping

**DOI:** 10.3390/biomimetics9120780

**Published:** 2024-12-21

**Authors:** Zhongjin Zhang, Zhitai Liu, Weiyang Lin, Wei Cheng

**Affiliations:** 1 Research Institute of Intelligent Control and Systems, Harbin Institute of Technology, Harbin 150001, China; zjzhang@stu.hit.edu.cn (Z.Z.); ztliu@hit.edu.cn (Z.L.); 2 Devol Advanced Automation, Inc., Shenzhen 518101, China

**Keywords:** direct-drive system, parameter adaptive, integral sliding mode observer, damping ratios, backstepping method, cogging force

## Abstract

Direct-drive servo systems are extensively applied in biomimetic robotics and other bionic applications, but their performance is susceptible to uncertainties and disturbances. This paper proposes an adaptive disturbance rejection Zeta-backstepping control scheme with adjustable damping ratios to enhance system robustness and precision. An iron-core permanent magnet linear synchronous motor (PMLSM) was employed as the experimental platform for the development of a dynamic model that incorporates compensation for friction and cogging forces. To address model parameter uncertainties, an indirect parameter adaptation strategy based on a recursive least squares algorithm was introduced. It updates parameters based on the system state instead of output error, ensuring robust parameter convergence. An integral sliding mode observer (ISMO) was constructed to estimate and compensate for residual uncertainties, achieving finite-time state estimation. The proposed Zeta-backstepping controller enables adjustable damping ratios through parameterized control laws, offering flexibility in achieving desired dynamic performance. System stability and bounded tracking performance were validated via a second-order Lyapunov function analysis. Experimental results on a real PMLSM platform demonstrated that, while achieving adjustable damping ratio dynamic characteristics, there is a significant improvement in tracking accuracy and disturbance suppression. This underscores the scheme’s potential for advancing precision control in biomimetic robotics and other direct-drive system applications.

## 1. Introduction

Direct-drive servo systems have found widespread application in the fields of bionics and biomimetic robotics due to their high thrust, fast response, and high positioning accuracy [1,2,3,4]. Bionic systems and robots often need to replicate complex biological motion patterns, achieving precise and flexible motion control to meet the demands of high-end manufacturing, medical rehabilitation, and intelligent services [5,6]. However, the performance of these systems relies heavily on the precise control of servo systems, particularly in scenarios requiring the management of nonlinear dynamic characteristics, external disturbances, and high-precision motion control [7]. Therefore, conducting in-depth research on the precise and effective control of direct-drive servo systems is not only crucial for addressing practical application bottlenecks in the field of bionics but also serves as an essential foundation for advancing biomimetic robotics technology.

To improve the control performance of direct-drive servo systems, various advanced control algorithms have been developed to address issues such as parameter perturbations, unknown disturbances, and system uncertainties. For instance, a PID controller optimized using a genetic algorithm was proposed for direct-drive servo systems in [8], effectively improving the system’s dynamic response characteristics. In [9], an adaptive backstepping-based speed tracking controller was designed for permanent magnet synchronous motor (PMSM) systems, addressing parameter uncertainties and external disturbances. The feedback linearization technique was applied in [10] to develop a robust controller for linear PMSMs, significantly enhancing the system’s anti-disturbance performance. Adaptive robust control, which combines the benefits of adaptive and robust control strategies, can effectively handle parameter variations and disturbances [11]. This method has been widely applied to PMSM systems [12] and electro-hydraulic servo systems [13]. Furthermore, Ref. [14] proposed a linear/nonlinear active disturbance rejection control (ADRC) switching strategy, achieving accurate and robust speed control for PMSMs. Although these methods have improved control performance, they often rely on nonlinear force models for feedforward compensation and require high feedback control gains to suppress uncertainties and maintain performance. However, under varying operating conditions or in the presence of external disturbances, control performance may degrade, potentially leading to instability.

Observer-based disturbance estimation methods have gained significant attention for ensuring robust disturbance rejection and maintaining control performance [15]. Various techniques have been developed, including equivalent-input-disturbance observers [16], adaptive disturbance observers (ADO) [17], high-gain observers [18], nonlinear disturbance observers [19,20], extended state observers (ESO) [21,22,23], sliding mode disturbance observers (SMDO) [24,25,26], and filter-based observers [27]. Despite these advancements, several challenges persist. Many approaches assume the Lipschitz condition, which often fails in practical servo systems due to discontinuous friction. Additionally, the requirement for disturbance derivatives, commonly present in these methods, is overly restrictive and unsuitable for managing the complex disturbances encountered in practical systems. The development of artificial neural network (NN) methods has introduced intelligent estimation and compensation algorithms, including radial basis function neural networks (RBFNN) [28], probabilistic fuzzy NN approximators [29], and B-spline wavelet NN observers [30]. However, these approaches frequently involve complex designs, demand significant computational resources, and have limited practical applicability. Among the various methods, sliding mode observers are distinguished by their capability to bypass the Lipschitz condition and the assumption of differentiable disturbances, delivering strong estimation performance with relatively simple implementation. To overcome the limitation of traditional sliding mode observers in ensuring invariance during the reaching phase, an integral sliding mode approach was introduced in [31], ensuring global sliding mode invariance. Additionally, an integral sliding mode observer was presented within the adaptive robust control framework [32], significantly enhancing the disturbance rejection performance of linear motors.

The aforementioned studies mainly focus on improving steady-state performance and disturbance rejection. To enhance transient performance, methods such as funnel control [33], prescribed performance control [34], and barrier function techniques [35] have been developed. These approaches transform error states or use reciprocal nonlinear gain to convert constrained error control into bounded control of transformed states. While these methods are effective in regulating both transient and steady-state performance, they primarily rely on feedback suppression for managing overshoot, an important metric in transient performance. Notably, few methods actively regulate overshoot. To fill this gap, the Zeta-backstepping method was proposed in [36], enabling explicit damping ratio adjustment through prescribed rules, thus facilitating active overshoot control.

Based on the preceding discussion, this paper proposes a damping ratio-adjustable adaptive disturbance rejection controller for direct-drive servo systems. By employing the Zeta-backstepping method, the controller enables the system’s damping ratio to be adjusted during positioning and transient tracking, allowing for customizable overshoot control. To manage parameter uncertainties, a compensation model is developed for the system’s nonlinear dynamics, while an indirect adaptive strategy is employed for parameter estimation to ensure convergence. For residual uncertainties and disturbances, an integral sliding mode observer is used to estimate and compensate for disturbances without assuming differentiability, making it suitable for complex, real-world conditions. Using the second-order Lyapunov method, it is demonstrated that the system output, with adjustable damping, achieves partial stability. The proposed approach is experimentally validated on an iron-core permanent magnet synchronous linear motor, showcasing its effectiveness and superior performance.

The main contributions of this study are as follows:

1. Accurate modeling and compensation of primary nonlinear forces in the direct-drive linear motor system. Parameter uncertainties and unknown disturbances are addressed through an indirect adaptive strategy and an integral sliding mode observer, respectively.
2. Introduction of a recursive least-squares with forgetting factor (FFRLS) algorithm to establish the adaptive parameter estimation law. This approach, driven by system input and output states rather than output error, ensures effective parameter convergence and decouples parameter estimation from the controller design.
3. A disturbance observer that does not require differentiable disturbances, broadening its applicability. This observer achieves precise state estimation within finite time, enhancing robustness against system disturbances.
4. The Zeta-backstepping controller ensures system stability and provides a tuning rule for damping ratio adjustment, enabling explicit overshoot control during both positioning and transient tracking phases.

The remainder of this paper is structured as follows. Section 2 offers a detailed description of the system, using a permanent magnet linear synchronous motor (PMLSM) as a representative example of direct-drive servo systems, including friction and cogging force modeling. Section 3 outlines the controller design process, covering parameter adaptation, observer estimation, and the Zeta-backstepping damping adjustment strategy. Section 4 presents the experimental results conducted on an iron-core PMLSM, validating the effectiveness and performance advantages of the proposed control scheme. Finally, conclusions are summarized in Section 5.

## 2. System Description

Direct-drive systems, which eliminate intermediate mechanical transmission components, directly drive the inertial load using a servo electric driver. This paper addresses the challenges of positioning and tracking control in direct-drive servo systems by designing and implementing a damping ratio-adjustable adaptive robust disturbance rejection controller.

This study focuses on an iron-core permanent magnet linear synchronous motor (PMLSM) system, which is widely employed in high-end manufacturing due to its high thrust density and superior thermal performance. These characteristics make it ideal for high-acceleration, heavy-load precision applications. However, the presence of nonlinear effects such as friction and cogging forces poses significant challenges for achieving precise motion control. To mitigate these issues, developing an appropriate nonlinear model proves effective for compensating these forces.

Let $\ddot{q}$, $\dot{q}$, and *q* represent the acceleration, velocity, and position of the linear motor, respectively. The dynamic model of the system can be expressed as:

(1) $$M\ddot{q}=k_{u}u\left(\nu \right)-F_{f}\left(\dot{q}\right)-F_{c}\left(q\right)+F_{d}\left(q,\dot{q},t\right)$$

where *M* represents the mass of the inertial load, $k_{u}$ is the motor torque constant, and u(ν) denotes the control input, defined as $u\left(\nu \left(t\right)\right)=\mathrm{sign}\left(\nu \right)\min\{|\nu |,u_{M}\}$. Here, ν represents the control command and $u_{M}$ is the maximum allowable input to the actuator. The terms $F_{f}$, $F_{c}$, and $F_{d}$ represent the friction force, cogging force, and unknown disturbances, respectively.

Friction, a common nonlinear phenomenon in servo systems, primarily consists of viscous resistance and Coulomb friction. The Coulomb-viscous friction model, used for compensating these forces, is defined as:

(2) $$F_{f}\left(\dot{q}\right)=B\dot{q}+A_{c}S_{c}\left(\dot{q}\right)$$

where *B* and $A_{c}$ are the viscous and Coulomb friction coefficients, respectively, and $S_{c}\left(\dot{q}\right)$ is the Coulomb friction function, often approximated by a smooth function such as $\frac{\pi }{2}\arctan\left(900\dot{q}\right)$ to replace the discontinuous signum function $\mathrm{sign}\left(\dot{q}\right)$.

The cogging force, a significant disturbance in iron-core PMLSM, limits control performance by causing thrust ripple due to the interaction between the stator’s permanent magnets and the mover’s iron core. Owing to its periodic nature, the cogging force is typically modeled as:

(3) $$F_{c}\left(q\right)=A_{r}^{T}S_{r}\left(q\right)=\sum_{k=1}^{n}A_{rk}\sin\left(\omega_{k}q+\phi_{k}\right)$$

where $A_{r}={\left[A_{r1},\ldots ,A_{rn}\right]}^{T}\in R^{n}$, and $S_{r}\left(q\right)={\left[\sin\left(\omega_{1}q+\phi_{1}\right),\ldots ,\sin\left(\omega_{n}q+\phi_{n}\right)\right]}^{T}$ is the basis function vector. $A_{rk}$, $\omega_{k}$, and $\phi_{k}$ represent the amplitude, frequency, and initial phase of the *k*-th harmonic component, respectively, while *n* denotes the number of harmonic components.

By combining the friction model (2) and the cogging force model (3), the system’s dynamic model (1) can be expressed in the state-space form as:

(4) $$\begin{array}{cc} {\dot{x}}_{1} & =x_{2}\\ {\dot{x}}_{2} & =\theta_{1}u\left(\nu \right)-\theta_{2}x_{2}-\theta_{3}S_{c}\left(x_{2}\right)-\theta_{4}^{T}S_{r}\left(x_{1}\right)+d \end{array}$$

where $x={\left[x_{1},x_{2}\right]}^{T}={\left[q,\dot{q}\right]}^{T}$ is the state vector, and $\theta ={\left[\theta_{1},\theta_{2},\theta_{3},\theta_{4}^{T}\right]}^{T}$ is the parameter vector defined as: $\theta_{1}=\frac{k_{u}}{M}$, $\theta_{2}=\frac{B}{M}$, $\theta_{3}=\frac{A_{c}}{M}$, and $\theta_{4}={\left[\frac{A_{r1}}{M},\ldots ,\frac{A_{rn}}{M}\right]}^{T}={\left[\theta_{4,1},\ldots ,\theta_{4,n}\right]}^{T}$. $d=\frac{F_{d}}{M}$ represents the normalized disturbance.

The control objective is to design a system controller with an adjustable damping ratio to regulate the control input u(ν) such that the system output accurately tracks the reference signal $x_{d}\left(t\right)$ accurately while explicitly managing overshoot during transient phases. To facilitate the controller design, the following assumptions are introduced:

**Assumption** **A1.**  *System parameter uncertainties and unknown disturbances are bounded within known ranges:*

(5) $$\theta \in \Omega_{\theta }≜\left\{\theta_{i}\;|\;\theta_{i\min}\leq \theta_{i}\leq \theta_{i\max}\right\},\;d\in \Omega_{d}≜\left\{d\;|\;\left|d\right|\leq \delta_{d}\right\}$$

*where $i=\left\{1,2,\ldots ,n+3\right\}$. $\theta_{\min}={\left[\theta_{1\min},\ldots ,\theta_{(3+n)\min}\right]}^{T}$, $\theta_{\max}={\left[\theta_{1\max},\ldots ,\theta_{(3+n)\max}\right]}^{T}$ denote the minimum and maximum values of θ, respectively, and they are known constant vectors. $\delta_{d}>0$ is also a known constant.*

**Assumption** **A2.**  *The system’s kinetic energy are constrained:*

(6) $$Mx_{2}^{2}\leq E_{k},\;E_{k}\in R^{+}.$$

*It can be deduced from the above assumption that the velocity state of the system is bounded, i.e.,*

(7) $$\left|x_{2}\right|\leq \sqrt{E_{k}/M_{\min}},\;M_{\min}=\min\left(M\right).$$

## 3. Control System Design

In this section, the parameter uncertainties and unknown disturbances in the PMLSM system are addressed using an indirect adaptive strategy and an integral sliding mode observer, respectively, for estimation and compensation. Additionally, a tracking controller with an adjustable damping ratio is synthesized based on the Zeta-backstepping design method, along with corresponding damping ratio adjustment rules.

### 3.1. Parameter Estimation

The core of adaptive model compensation lies in establishing an online parameter adaptation law, guided by prior information and based on the system’s dynamic model (4), to address nonlinear effects in the system [37]. To ensure that parameter estimates remain within known bounds, as specified in Assumption 1, the following discontinuous projection mapping function is applied:

(8) $${\mathrm{Proj}}_{\hat{\theta }}\left(•\right)=\left\{\begin{array}{cc} •, & \hat{\theta }\in {\bar{\Omega }}_{\theta }\;\mathrm{or}\;\eta_{\hat{\theta }}^{T}•\leq 0\\ \left(I-\frac{\Gamma \eta_{\hat{\theta }}\eta_{\hat{\theta }}^{T}}{\eta_{\hat{\theta }}^{T}\Gamma \eta_{\hat{\theta }}}\right)•, & \hat{\theta }\in \partial \Omega_{\theta }\;\mathrm{and}\;\eta_{\hat{\theta }}^{T}•>0, \end{array}\right.$$

where $\hat{\theta }$ is the estimated parameter value, ${\bar{\Omega }}_{\theta }$ and $\partial \Omega_{\theta }$ denote the interior and boundary of the set $\Omega_{\theta }$, respectively, and $\eta_{\hat{\theta }}$ is the outward unit normal vector for $\hat{\theta }\in \partial \Omega_{\theta }$.

To decouple parameter estimation from controller design effectively, a saturation function is introduced to constrain the adaptive rate with a predefined upper limit:

(9) $${\mathrm{sat}}_{{\dot{\theta }}_{M}}\left(•\right)=\mathrm{sign}\left(•\right)\min\left\{∥•∥,{\dot{\theta }}_{M}\right\}$$

where ${\dot{\theta }}_{M}>0$ is the maximum allowable adaptive rate.

The adaptive parameter law is then expressed as:

(10) $$\dot{\hat{\theta }}={\mathrm{sat}}_{{\dot{\theta }}_{M}}\left({\mathrm{Proj}}_{\hat{\theta }}\left(\Gamma \chi \right)\right),\;\hat{\theta }\left(0\right)\in \Omega_{\theta },$$

where χ is the adaptation function, and Γ is the continuously differentiable, positive-definite adaptation rate matrix.

To design the parameter adaptation law, we initially consider only the model’s parameter uncertainties, setting d=0 to disregard disturbances, which will be later handled by the observer. Thus, the system dynamics (4) can be rewritten as the following parameter estimation model:

(11) $$y=\theta_{1}u\left(\nu \right)-\theta_{2}x_{2}-\theta_{3}S_{c}\left(x_{2}\right)-\theta_{4}^{T}S_{r}\left(x_{1}\right)=\varphi^{T}\theta$$

where $y={\dot{x}}_{2}$, $\varphi ={\left[u\left(\nu \right),-x_{2},-S_{c}\left(x_{2}\right),-S_{r}\left(x_{1}\right)\right]}^{T}$ is the parameter regressor.

By applying a stable low-pass filter $Q_{f}\left(•\right)$ to the model (11), one can obtain

(12) $$y_{f}=\varphi_{f}^{T}\theta =\theta_{1}u_{f}-\theta_{2}x_{2f}-\theta_{3}S_{cf}-\theta_{4}^{T}S_{rf}$$

where $y_{f}$ denotes the output of the filter $•Q_{f}\left(•\right)$ with $x_{2}$ as the input. Correspondingly, $u_{f}$, $x_{2_{f}}$, $S_{cf}$, and $S_{rf}$ represent the outputs of the filter $Q_{f}\left(•\right)$ for the inputs *u*, $x_{2}$, $S_{c}$, and $S_{r}$, respectively.

Define the model’s estimated output ${\hat{y}}_{f}$ and estimation error ϵ as follows:

(13) $${\hat{y}}_{f}=\varphi_{f}^{T}\hat{\theta },\;\epsilon ={\hat{y}}_{f}-y_{f}$$

leading to $\epsilon =\varphi_{f}^{T}\tilde{\theta }$, where $\tilde{\theta }=\hat{\theta }-\theta$ is the parameter estimation error. Based on this error model, we apply the recursive least squares with forgetting factor (FFRLS) algorithm for parameter estimation.

Thus, the adaptive function χ and adaptive rate matrix Γ in the adaptation law (10) are given by:

(14) $$\begin{array}{cc} \chi & =-\frac{\varphi_{f}\epsilon }{1+\gamma \varphi_{f}^{T}\Gamma \varphi_{f}}, \end{array}$$

(15) $$\begin{array}{cc} \dot{\Gamma } & =\left\{\begin{array}{cc} \alpha \Gamma -\frac{1}{1+\gamma \varphi_{f}^{T}\Gamma \varphi_{f}}\Gamma \varphi_{f}\varphi_{f}^{T}\Gamma ,\; & \mathrm{if}\;\lambda_{\max}\left(\Gamma \left(t\right)\right)\leq \rho_{M}\\ 0,\; & \mathrm{otherwise}, \end{array}\right. \end{array}$$

where γ>0 is a design constant, α is the forgetting factor, and $\rho_{M}$ specifies the maximum ∥Γ(t)∥ to prevent estimator saturation.

**Lemma** **1**  ([37])**.** *Following the saturation-constrained projection mapping adaptive law (10) and applying the recursive least squares estimator to determine the adaptive function (14) and adaptive rate (15), the prediction error ϵ defined in (13) can satisfy $\epsilon \in L_{2}\left(0,\infty \right)\cap L_{\infty }\left[0,\infty \right)$, and the parameter estimate error satisfies $\tilde{\theta }\in L_{2}\left[0,\infty \right)$.*

### 3.2. Observer Design

After compensating for parameter estimation, the system dynamics (4) can be rewritten as:

(16) $$\begin{array}{cc} {\dot{x}}_{1} & =x_{2}\\ {\dot{x}}_{2} & ={\hat{\theta }}_{1}u\left(\nu \right)-{\hat{\theta }}_{2}x_{2}-{\hat{\theta }}_{3}S_{c}\left(x_{2}\right)-{\hat{\theta }}_{4}^{T}S_{r}\left(x_{1}\right)+d_{l} \end{array}$$

where $d_{l}=-\varphi^{T}\tilde{\theta }+d$ represents the lumped uncertainty, encompassing both the parameter estimation residuals and external disturbances.

To effectively estimate and mitigate the lumped uncertainty $d_{l}$, we propose an integral sliding mode observer defined as:

(17) $$\begin{array}{cc} {\dot{\hat{x}}}_{1} & ={\hat{x}}_{2}-\iota_{1}\left({\hat{x}}_{1}-x_{1}\right)+\omega_{1}\\ {\dot{\hat{x}}}_{2} & ={\hat{\theta }}_{1}u\left(\nu \right)-{\hat{\theta }}_{2}x_{2}-{\hat{\theta }}_{3}S_{c}\left(x_{2}\right)-{\hat{\theta }}_{4}^{T}S_{r}\left(x_{1}\right)+\iota_{2}\omega_{1}+\omega_{2} \end{array}$$

where ${\hat{x}}_{1}$ and ${\hat{x}}_{2}$ are the estimates of the states $x_{1}$ and $x_{2}$, respectively. $\iota_{1}$ and $\iota_{2}$ are positive observer gain constants to be determined, $\omega_{1}$ and $\omega_{2}$ are the observer inputs defined as follows.

(18) $$\begin{array}{cc} \omega_{1} & =-\eta_{o}\mathrm{sign}\left(\xi_{1}\left(t\right)\right)-\left(\varrho_{o}+\left|{\hat{x}}_{2}\right|\right)\mathrm{sign}\left(s\left(t\right)\right) \end{array}$$

(19) $$\begin{array}{cc} \omega_{2} & =\kappa_{o}\mathrm{sign}\left(\omega_{1}\left(t\right)\right) \end{array}$$

where $\xi_{1}={\hat{x}}_{1}-x_{1}$ is the estimation error for state $x_{1}$. $\eta_{o}$, $\varrho_{o}$, and $\kappa_{o}$ are tunable design parameters, and s(t) represents the integral sliding mode variable designed as follows:

(20) $$s\left(t\right)=\xi_{1}\left(t\right)+\int_{0}^{t}\left(\eta_{o}\mathrm{sign}\left(\xi_{1}\left(\tau \right)\right)+\iota_{1}\xi_{1}\left(\tau \right)\right)d\tau -\xi_{1}\left(0\right)$$

where $\xi_{1}\left(0\right)={\hat{x}}_{1}\left(0\right)-x_{1}\left(0\right)$ denotes the initial value of state estimation error $\xi_{1}\left(t\right)$.

Letting $\xi_{2}={\hat{x}}_{2}-x_{2}$ and combining the modified system model (16) with the observer model (17), we derive the dynamics of the state estimation errors as follows:

(21) $$\begin{array}{cc} {\dot{\xi }}_{1} & =\xi_{2}-\iota_{1}\xi_{1}+\omega_{1}, \end{array}$$

(22) $$\begin{array}{cc} {\dot{\xi }}_{2} & =\iota_{2}\omega_{1}+\omega_{2}-d_{l}. \end{array}$$

**Theorem** **1.**  *Based on the sliding mode equivalent control theory [31], by selecting the integral sliding mode variable as defined in (20) and establishing the disturbance observer (17) while ensuring that the observer parameters satisfy the following conditions:*

(23) $$\eta_{o}>0,\;\varrho_{o}>\sqrt{E_{k}/M_{\min}},\;\kappa_{o}>\delta_{h},\;\iota_{1},\iota_{2}>0,$$

*where $\delta_{h}>∥\varphi ∥∥\theta_{M}∥+\delta_{d}\geq \left|d_{l}\right|$ is a positive constant with $\theta_{M}=\theta_{\max}-\theta_{\min}$. Then the state estimation errors $\xi_{1}$ and $\xi_{2}$ will converge to the zero equilibrium in finite time, i.e.,*

(24) $$\begin{array}{cc} \xi_{1} & =0,\;{\dot{\xi }}_{1}=0,\;\forall t\geq t_{1} \end{array}$$

(25) $$\begin{array}{cc} \xi_{2} & =0,\;{\dot{\xi }}_{2}=0,\;\forall t\geq t_{2} \end{array}$$

*where $0<t_{1},t_{2}<+\infty$. Additionally, the estimated value of the lumped uncertainty in the system can be expressed as:*

(26) $${\hat{d}}_{l}=\iota_{2}\omega_{1}+\omega_{2}.$$

The proof of Theorem 1 is given in the Appendix A.

The integral sliding mode observer effectively estimates the system’s unknown uncertainties and disturbances. However, the estimation results, derived from observer inputs $\omega_{1}$ and $\omega_{2}$, include sign functions, which inevitably introduce chattering in the estimated values. To ensure effective compensation with disturbance estimation, it is crucial to suppress these chattering to maintain overall system stability. To achieve this, we introduce a continuous boundary layer function, defined as $S_{\mathrm{con}}\left(•\right)=\frac{•}{|•|+\varepsilon }$, to approximate the sign function, where ε>0 is a constant that determines the width of the boundary layer. Additionally, to ensure the smoothness of the disturbance estimates, we apply a low-pass filter given by $F\left(s\right)=\frac{1}{\tau s+1}$. This filter further mitigates the effects of high-frequency switching in the observer input signals.

However, it is important to note that while the introduction of the boundary layer function and filter enhances the smoothness of the estimation results, it may also reduce the accuracy and robustness of the disturbance estimates. Therefore, a trade-off must be established between estimation performance and oscillation levels, necessitating careful determination of the parameters ε and τ based on the specific application requirements.

**Remark** **1.**  *The designed integral sliding mode observer guarantees boundedness for both the state estimation error and the disturbance estimate. For $\xi_{1}$, boundedness is straightforward to establish via its finite-time convergence proof, with its derivative ${\dot{\xi }}_{1}$ remaining bounded per (A5). Since sliding mode invariance always holds, the observer inputs $\omega_{1}$, $\omega_{2}$ defined in (18) and (19) are thus bounded. Consequently, from (21) and (26), both $\xi_{2}$ and the disturbance estimate ${\hat{d}}_{l}$ remain bounded. Therefore, there exists a positive constant $\delta_{o}$ such that $\left|{\hat{d}}_{l}\right|<\delta_{o}$ holds.*

### 3.3. Controller Synthesis

To achieve controllable damping ratio for system output, the Zeta-backstepping method is employed in the controller design. This innovative approach stands apart conventional backstepping by not only ensuring system stability but also providing an explicit tuning law for achieving desired damping ratios. This control method thus allows for direct management of overshoot characteristics and system dynamic response.

Define the output tracking error as $z_{1}=x_{1}-x_{d}$, and let $z_{2}=x_{2}-\alpha_{1}$, where $\alpha_{1}$ is a virtual control variable. The time derivative of $z_{1}$ can then be expressed as

(27) $${\dot{z}}_{1}=z_{2}+\alpha_{1}-{\dot{x}}_{d}.$$

Unlike traditional backstepping methods, where the virtual control variable $\alpha_{1}$ is typically chosen as $\alpha_{1}=-k_{1}z_{1}+{\dot{x}}_{d}$, the Zeta-backstepping approach simplifies it to

(28) $$\alpha_{1}={\dot{x}}_{d}.$$

Substituting (28) into (27) yields:

(29) $${\dot{z}}_{1}=z_{2}.$$

Taking the derivative of $z_{2}$ and incorporating the system dynamics from (16), one obtains

(30) $${\dot{z}}_{2}={\hat{\theta }}_{1}u\left(\nu \right)-{\hat{\theta }}_{2}x_{2}-{\hat{\theta }}_{3}S_{c}\left(x_{2}\right)-{\hat{\theta }}_{4}^{T}S_{r}\left(x_{1}\right)+d_{l}-{\dot{\alpha }}_{1}.$$

Following the Zeta backstepping method, we design the control input *u* as

(31) $$\begin{array}{c} u\left(\nu \right)=\mathrm{sign}\left(\nu \right)\min\{|\nu |,u_{M}\},\\ \nu =\frac{1}{{\hat{\theta }}_{1}}\left(-k_{1}z_{1}-k_{2}z_{2}+{\hat{\theta }}_{2}x_{2}+{\hat{\theta }}_{3}S_{c}\left(x_{2}\right)+{\hat{\theta }}_{4}^{T}S_{r}\left(x_{1}\right)-{\hat{d}}_{l}+{\dot{\alpha }}_{1}\right), \end{array}$$

where $k_{1},k_{2}>0$ are feedback control gains that dictate the system’s damping ratio.

Substituting the control law (31) into (30) results in

(32) $${\dot{z}}_{2}=-k_{1}z_{1}-k_{2}z_{2}-{\tilde{d}}_{l},$$

where ${\tilde{d}}_{l}={\hat{d}}_{l}-d_{l}$.

**Theorem** **2.**  *For the iron-core PMLSM system described by (4), with uncertainty compensation through the parameter adaptation law (10) and the integral sliding mode observer (17), the proposed controller (31) guarantees practical stability of the system tracking error with an adjustable damping ratio ζ. Specifically:*

*1.*  *If the control gains satisfy $k_{1}=\beta^{2}$ and $k_{2}=2\beta$ with β>0 as a design parameter, the system tracking error can achieve an approximately critically damped response, i.e., ζ≈1.*
*2.*  *If the control gains satisfy $k_{1}=\beta_{1}^{2}+\beta_{2}^{2}$ and $k_{2}=2\beta_{2}$ with $\beta_{1},\beta_{2}>0$ as design parameters, then the system tracking error exhibits an approximately underdamped response, where the damping ratio $\zeta \approx \frac{\beta_{1}}{\sqrt{\beta_{1}^{2}+\beta_{2}^{2}}}$.*

**Proof.**  To determine stability and convergence, we choose $z_{1}$ as the variable for the Lyapunov function and define $V_{1}=z_{1}$. We then construct a second-order Lyapunov function as follows:

(33) $$V={\ddot{V}}_{1}+k_{2}{\dot{V}}_{1}+k_{1}V_{1}.$$

Considering the definition of $V_{1}$ and combining (29) and (32), one can obtain

(34) $$\left|V\right|=\left|{\ddot{V}}_{1}+k_{2}{\dot{V}}_{1}+k_{1}V_{1}\right|=\left|{\dot{z}}_{2}+k_{2}z_{2}+k_{1}z_{1}\right|=\left|{\tilde{d}}_{l}\right|\leq \delta ,$$

where $\delta =\delta_{h}+\delta_{o}$ represents a bound on the lumped disturbance terms.

When the system’s parameter uncertainties and unknown disturbances are adequately compensated, i.e., δ=0, the inequality (34) simplifies into a second-order homogeneous differential equation with the characteristic equation

(35) $$r^{2}+k_{2}r+k_{1}=0$$

The corresponding discriminant is $\Delta =k_{2}^{2}-4k_{1}$. According to Lemma 2 in [36], it can be obtain that:

When Δ=0: The characteristic Equation (35) has a pair of identical real roots, denoted as −β with β>0. In this case, the relationships between the gain parameters are $k_{1}=\beta^{2}$ and $k_{2}=2\beta$. The solution of (34) is asymptotically stable with a damping ratio ζ=1, indicating a critically damped response.

When Δ<0: The characteristic Equation (35) yields a pair of complex conjugate roots, denoted as $-\beta_{1}\pm \beta_{2}i$ with $\beta_{1},\beta_{2}>0$. The gain parameter relationships are $k_{1}=\beta_{1}^{2}+\beta_{2}^{2}$ and $k_{2}=2\beta_{2}$. The system remains asymptotically stable, and the damping ratio satisfies $0<\zeta =\frac{\beta_{1}}{\sqrt{\beta_{1}^{2}+\beta_{2}^{2}}}<1$, corresponding to an underdamped response.

On the other hand, if parameter uncertainties and unknown disturbances persist, i.e., δ>0, inequality (34) becomes a second-order nonhomogeneous differential equation. By establishing boundary conditions and applying the comparison principle, it can be demonstrated that the solution to inequality (34) remains bounded.

By using the characteristic Equation (35) and selecting either real root solutions or complex conjugate root solutions, the inequality (34) can be satisfied, ensuring the stability of the variable $V_{1}$. In this scenario, even with δ>0, the solution of (34) still closely approximates the response characteristics for δ=0, thereby maintaining similar damping behavior. □

## 4. Experimental Results

### 4.1. Experimental Platform

**Figure 1:**
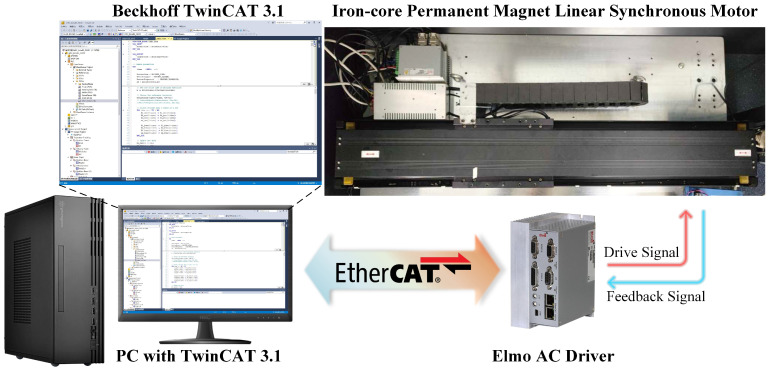
Iron-core permanent magnet linear synchronous motor platform.

To validate the effectiveness and advantages of the proposed control scheme, an iron-core permanent magnet linear synchronous motor (PMLSM) testing platform was established, as shown in Figure 1. The control algorithm was implemented in the TwinCAT 3.1 environment on a computer, which was connected to the servo drive via a standard RJ45 Ethernet cable. The platform featured a Yaskawa SGLFW-50A200B iron-core F-type linear motor, operated using an Elmo G-OBO13/230FEHN1 AC servo drive. Position measurement was performed with a Renishaw RGS20 linear scale coupled with an RGH22B readhead. Details of these devices are given in Table 1. The servo drive is powered by a 220V AC supply and connected to the linear motor via a three-phase AC power cable. Upon receiving current commands, it generates the corresponding current output to drive the motor. The linear scale measures the position of the mover and outputs quadrature differential sine signals to the drive for feedback.

**Figure 2:**
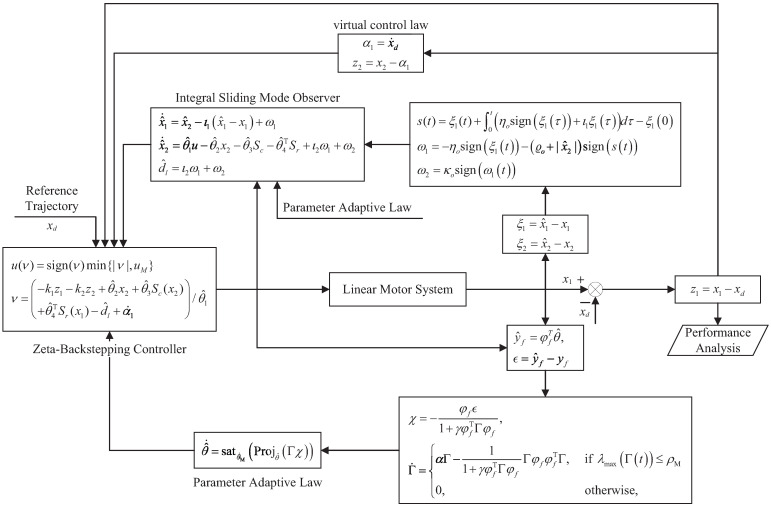
Diagram of the proposed controller implementation in TwinCAT.

To implement the proposed control strategy, the dynamics of the PMLSM, including the modeling of friction and cogging forces, were described in Equations (1)–(7) in Section 2, forming the basis for parameter estimation (10)–(15), disturbance observer design (17)–(20), and controller synthesis (31) in Section 3. Specifically, the control algorithm was implemented using Structured Text (ST) language in TwinCAT software (version 4024.60), with the main equations and their relationships illustrated in Figure 2. Motion trajectory planning and current command generation were carried out within the TwinCAT environment, with the drive configured in Cyclic Synchronous Torque (CST) mode. The EtherCAT communication combined with the CiA 402 protocol, was employed to transmit control commands (*u*) from the TwinCAT software to the drive and upload feedback data ($x_{1},x_{2}$), achieving closed-loop control of the system. Both the program execution cycle and data sampling period were set to 1 ms.

### 4.2. Parameter Selection

**Figure 3:**
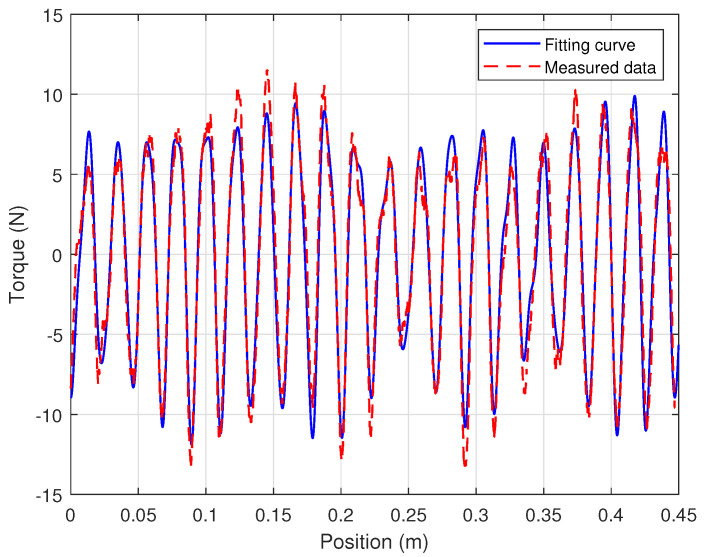
Measured data and fitting curve of cogging force.

**Figure 4:**
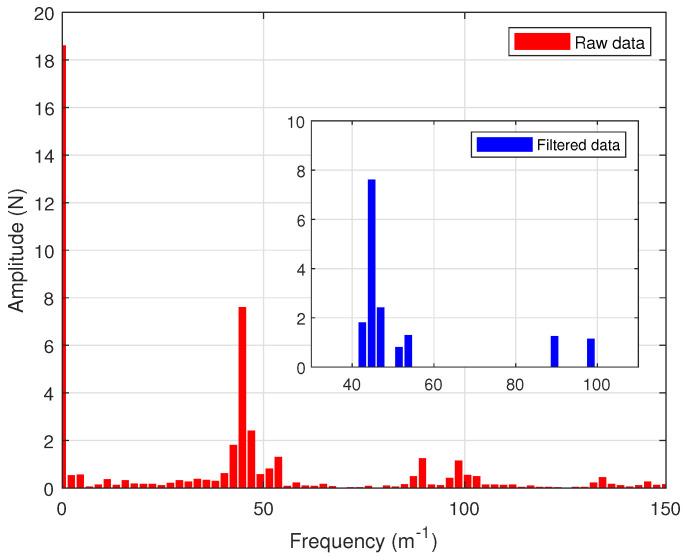
Fourier transform and processing of cogging force data.

Initial parameter estimates for the iron-core linear motor system were obtained through offline identification. The motor’s cogging force was measured following the method outlined in [38]. Neglecting the external disturbances in system (1), i.e., $F_{d}=0$, the linear motor was operated at a low constant speed. Under these conditions, $k_{u}u=F_{f}+F_{c}$, meaning the output torque ($k_{u}u$) of the linear motor can be regarded as the sum of the friction force $F_{f}$ and the cogging force $F_{c}$. The relationship between the motor’s output torque and the mover’s position can be obtained during constant-speed motion, as shown by the red dashed line in Figure 3. A Fast Fourier Transform (FFT) was performed on the measured force signal to obtain its spectral characteristics with respect to the motor’s displacement, as shown in Figure 4. The DC component in the spectrum (i.e., the component near 0 m^−1^) was considered the friction force, while the remaining components were attributed to the cogging force. To characterize the cogging force, the seven harmonic components with the largest amplitudes were selected from the spectrum, as illustrated in the subplot of Figure 4. These selected components were then subjected to an inverse Fourier transform to obtain time-domain data, which were used to identify the parameters of the cogging force model in (3). The identified model parameters are presented in Table 2, and the fitted curve is shown as the blue solid line in Figure 3.

The linear motor operates under no-load conditions, with the inertial load consisting only of the mover itself. The experimental control objective is to ensure that the motor mover can quickly and accurately track the desired trajectory $x_{d}$ while maintaining the specified damping ratio characteristics. Consequently, the parameter vector θ for the system model (4) comprises 10 components, with their initial estimates $\theta_{0}$, maximum $\theta_{\max}$, and minimum values $\theta_{\min}$ detailed in Table 3. For the indirect adaptive strategy, the remaining parameters were set as follows: γ=0.1, α=0.02, ${\dot{\theta }}_{M}=5000$, $\rho_{M}=50,000$, and $u_{M}=10$.

### 4.3. Experimental Design

In this section, we compare four control algorithms:

C1: Zeta-backstepping controller based on an indirect sliding mode observer (ISMO) without dependency on system models. This method relies solely on the ISMO for estimating and compensating for system uncertainties, without explicit friction or cogging force compensation.

C2: Builds upon C1 by incorporating a friction compensation model as defined in (2) while excluding cogging force compensation. It also uses the indirect adaptive strategy (10) for real-time online parameter estimation.

C3: Indirect adaptive robust controller (IARC) [32] based on the developed dynamic model (1), including friction (2) and cogging force (3) compensation. This approach employs the indirect parameter adaptive strategy (10)–(15) and synthesizes the controller using the traditional backstepping method. Additionally, it suppresses disturbances through robust feedback without relying on the ISMO for estimation and compensation.

C4: The proposed adaptive disturbance rejection Zeta-backstepping controller that integrates both friction (2) and cogging force (3) compensation, as described in (31). This control scheme is designed to provide adjustable damping ratios during the positioning phase, ensuring precise trajectory tracking and robust disturbance rejection.

To ensure the rigor and fairness of experimental testing, all experiments were conducted with the mover starting from the same initial position, and the initial values of the same parameters were kept consistent. Additionally, two quantitative evaluation metrics of the tracking error *e* were introduced to analyze the performance of different methods in terms of tracking and disturbance rejection: the maximum absolute (MAX) value $e_{m}=\max\left\{|e\left(t\right)|\right\}$ and the root mean square (RMS) value $e_{r}=\sqrt{1/(t_{e}-t_{s})\int_{t_{s}}^{t_{e}}e^{2}\left(t\right)dt}$, where $t_{s}$ and $t_{e}$ denote the start and end times, respectively.

To compare and analyze the performance of the proposed methods in terms of adjustable damping ratios, tracking accuracy, and disturbance rejection, the following test cases were designed.

#### 4.3.1. Case 1: Square Wave Positioning

To verify the characteristic of setting the damping ratio during the positioning phase, this case sets the target trajectory as a square wave with an amplitude of 0.04 m and a period of 10 s. This allows us to observe the algorithm’s response characteristics to step signals and the overshoot phenomenon under different damping ratio settings.

According to the damping ratio setting rules described in Theorem 2, three sets of parameters were selected: (a) β=20, (b) $\beta_{1}=15,\;\beta_{2}=15$, (c) $\beta_{1}=10,\;\beta_{2}=17$. These lead to corresponding damping ratios and control gains of: (i) $\zeta =1,\;k_{1}=400,k_{2}=40$, (ii) $\zeta =0.707,k_{1}=450,\;k_{2}=30$, (iii) $\zeta =0.507,k_{1}=389,\;k_{2}=30$. Since the trajectory used does not effectively excite the parameter adaptation process, the initial value of the parameter adaptation rate matrix Γ is set to zero, rendering it ineffective. The ISMO parameters were configured with $\eta_{o}=0.1$, $\varrho_{o}=0.4$, $\kappa_{o}=15$, $\iota_{1}=20$, and $\iota_{2}=20$. The boundary layer function $S_{\mathrm{con}}\left(•\right)$ was applied to approximate the sign functions of $\xi_{1}$, *s*, and $\omega_{1}$ in (18)–(20) with corresponding $\varepsilon_{\xi_{1}}=0.001$, $\varepsilon_{s}=0.01$, and $\varepsilon_{\omega_{1}}=0.1$. The filter F(s) was set to τ=0.3. In addition, for C3 using the IARC method, with reference to the design in Ref. [32], the control gains are set as c=15, $k_{\sigma }=5$, $k_{s}=0.05$.

**Figure 5:**
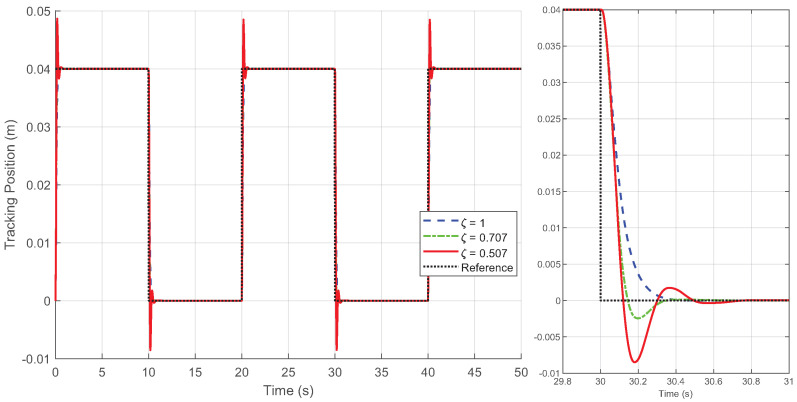
Position tracking response of the square wave trajectory under different damping ratio ζ for controller C1 in Case 1.

**Figure 6:**
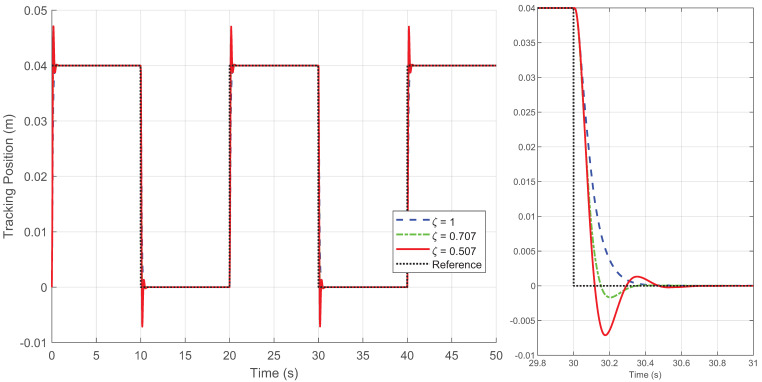
Position tracking response of the square wave trajectory under different damping ratio ζ for controller C2 in Case 1.

**Figure 7:**
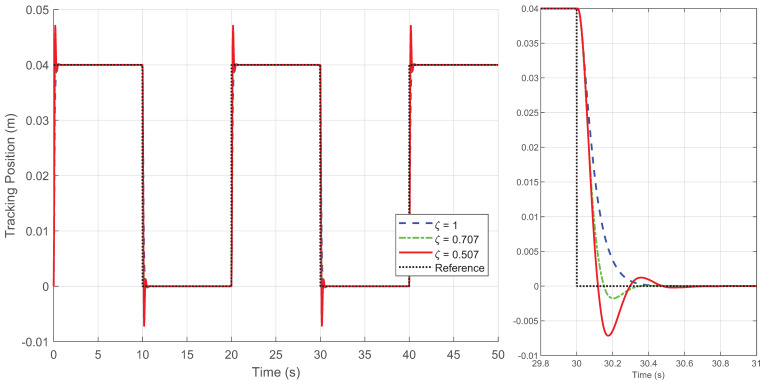
Position tracking response of the square wave trajectory under different damping ratio ζ for controller C4 in Case 1.

**Figure 8:**
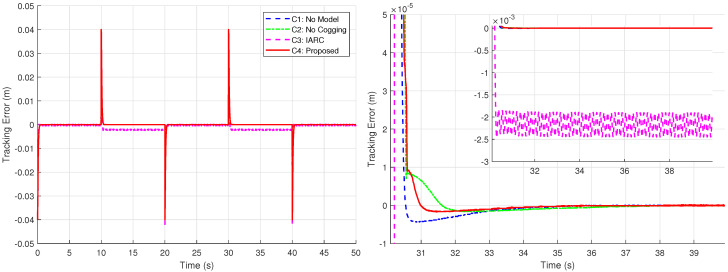
Position tracking errors of four controllers under the square wave trajectory (**left**) and local amplification of position tracking errors for a step input (**right**) when ζ=1 in Case 1.

**Figure 9:**
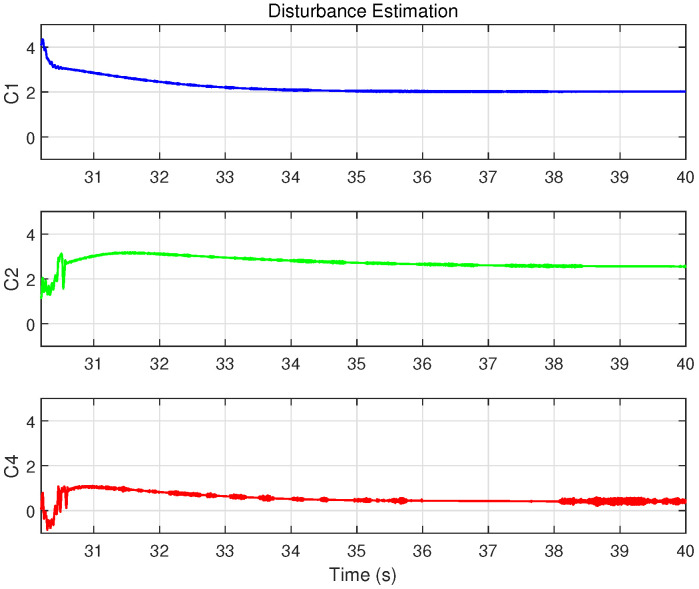
Disturbance estimation results of the controllers with ISMO for a single step input when the damping ratio is set to ζ=1 in Case 1.

By generating the desired square wave trajectory in TwinCAT as described and implementing the four control schemes through programming, the motion response of the linear motor was tested. The process data was recorded by TwinCAT, and the experimental results are presented in Figure 5, Figure 6, Figure 7, Figure 8 and Figure 9.

Figure 5, Figure 6 and Figure 7 display the response curves for controllers C1, C2, and C4 under damping ratios of ζ=1, 0.707, and 0.507, respectively. The results show that the overshoot behavior of each controller aligns closely with the specified damping ratio, confirming that the Zeta-backstepping controller can effectively control system overshoot via the prescribed damping ratio strategy. Notably, Figure 6 and Figure 7 reveal that the tracking curves of C2 and C4 are nearly indistinguishable under the current positioning motion conditions. This similarity arises from the minimal influence of the additional cogging force compensation in C4 relative to C2 in this scenario. However, subtle differences between the two are evident in the magnified view of the tracking error in Figure 8, which provides a more detailed comparison of their respective tracking performances.

Figure 8 illustrates the tracking errors of the four controllers, where the damping ratio of C1, C2, and C4 is set to ζ=1. For the IARC controller C3, designed based on the conventional backstepping method, the control gain is set lower because the method fails to operate at higher gains. Moreover, C3 exhibits uncontrollable overshoot in its tracking error response, along with steady-state errors and chattering, rendering it unsuitable for this type of positioning task. In contrast, the controllers C1, C2, and C4, designed using the Zeta-backstepping approach, are functional for positioning tasks. The model-based controllers C2 and C4 effectively reduce overshoot without compromising the damping characteristics. Controller C4, which incorporates a cogging force model, achieves a better balance between overshoot control and tracking performance.

Figure 9 compares disturbance estimates generated by the integral sliding mode observer for each controller during a step response. Notably, the controller C4, which includes the cogging force model, yields the smallest disturbance estimate, indicating that a more comprehensive system model can alleviate the observer’s burden and enhance overall disturbance rejection.

In summary, this experiment demonstrates that the proposed control scheme outperforms the controller synthesized using the conventional backstepping method in adapting to point-to-point positioning tasks. It enables explicit control of overshoot by following the prescribed damping ratio adjustment rules. Additionally, the introduction of ISMO ensures robust positioning and tracking performance, even under scenarios with unknown or partially known system models, validating the versatility of the proposed approach.

#### 4.3.2. Case 2: Sinusoidal Tracking

To evaluate the tracking performance of the proposed control scheme for continuous trajectories, this case followed the initial positioning phase and then tracked a sinusoidal trajectory defined by $x_{d}\left(t\right)=0.02\left[2-\cos\left(\pi t\right)\right]$ (m). As Case 1 has verified the effectiveness of different schemes under varying damping ratio settings, this case focus on the performance under critical damping, with β=20 and corresponding gains $k_{1}=400,\;k_{2}=40$. The parameter settings of ISMO are the same as Case 1. Additionally, for the IARC controller C3, a control gain switching strategy is required to enable a successful startup during the initial positioning phase and ensure subsequent tracking performance. Specifically, the control gains are initially set as c=35 and $k_{\sigma }=5$. After 5 s, they are adjusted to c=80 and $k_{\sigma }=18$, while $k_{s}$ remains fixed at 0.05 throughout the operation.

**Figure 10:**
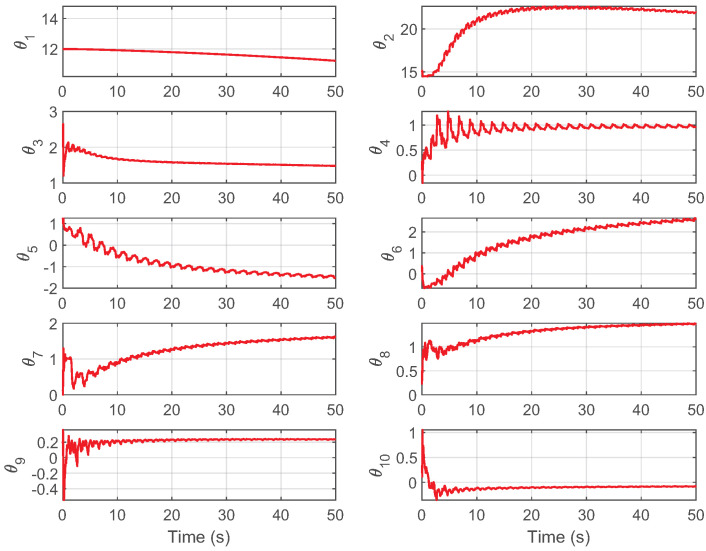
The results of online estimation of model parameters for controller C4 in Case 2.

**Figure 11:**
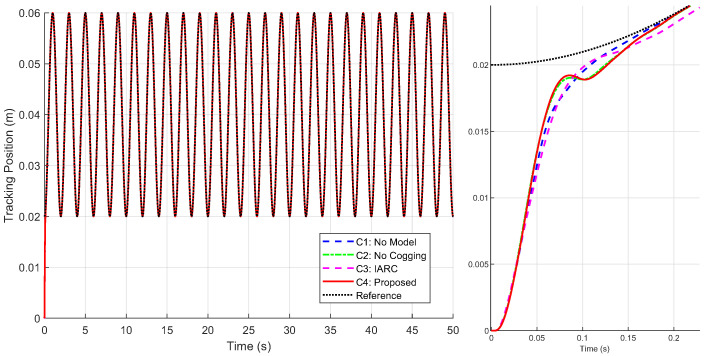
Position tracking response of four controllers under the sinusoidal trajectory (**left**) and local amplification of position tracking during the initial positioning phase (**right**) in Case 2.

**Figure 12:**
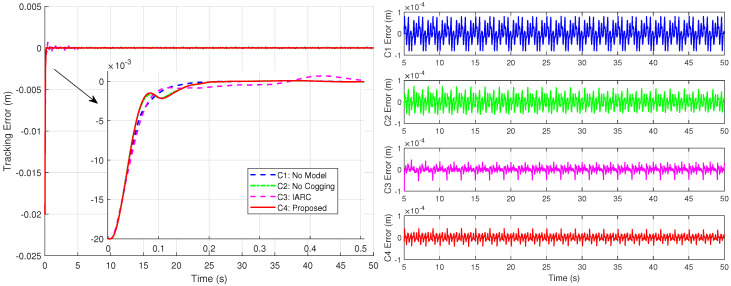
Position tracking errors under the sinusoidal trajectory with local amplification for initial positioning phase (**left**) and position tracking errors during trajectory tracking phase (**right**) in Case 2.

**Figure 13:**
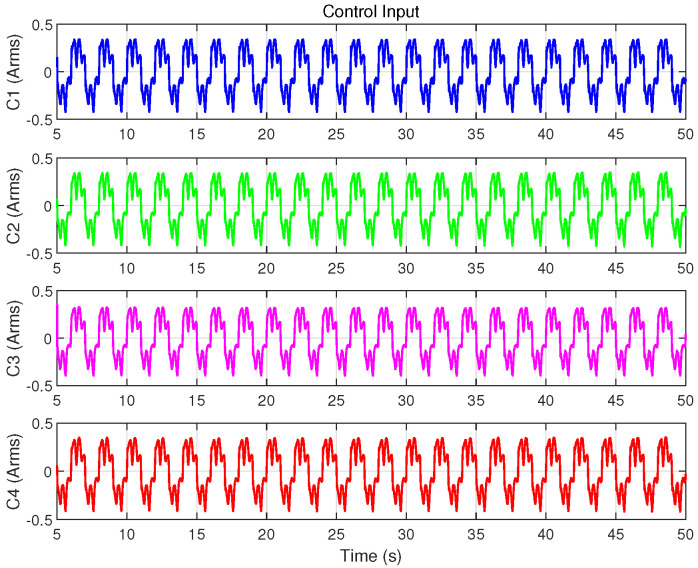
Control input of the four controllers in Case 2.

**Figure 14:**
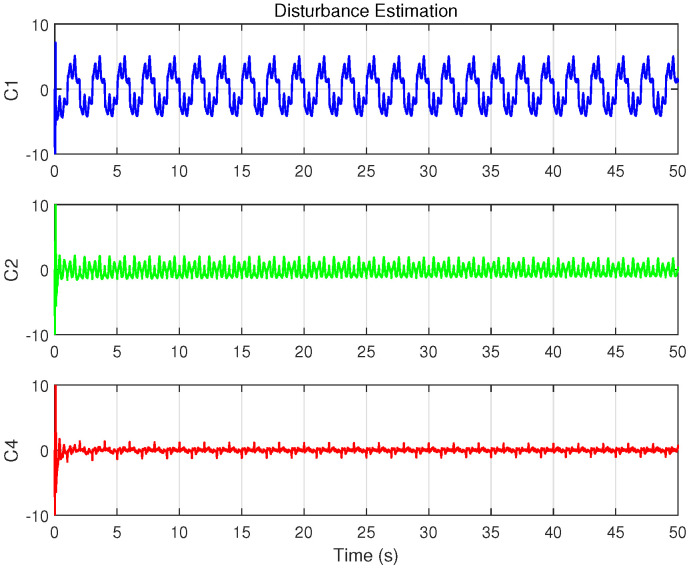
Disturbance estimation results of the controllers with ISMO in Case 2.

Similar to Case 1, the continuous sinusoidal trajectory $x_{d}\left(t\right)$ and the control algorithms are pre-programmed in TwinCAT, where the servo driver handles the conversion of control commands and uploads feedback data. Experimental data is recorded within the TwinCAT software, and the corresponding results are shown in Figure 10, Figure 11, Figure 12, Figure 13 and Figure 14 and Table 4.

Figure 10 illustrates the online parameter estimation for system model (4), demonstrating that the designed indirect adaptive strategy successfully promotes parameter convergence.

Figure 11 presents the position tracking responses of the four controllers to the sinusoidal trajectory, including the initial positioning phase, while Figure 12 shows the corresponding position tracking errors. It should be noted that, as validated in Case 1, C3 is unsuitable for positioning tasks. To ensure successful initialization, smaller gains were employed initially, which were subsequently switched to larger gains to achieve the tracking performance shown in the figures. In contrast, C1, C2, and C4 completed both initialization and tracking with a single set of gains while maintaining robust performance. During the sinusoidal trajectory tracking, controllers C2, C3 and C4 exhibit smaller tracking errors compared to C1, due to the inclusion of system model compensation and online parameter estimation, with errors decreasing over time. Since C4 further incorporated cogging force compensation based on C2, it achieves lower tracking errors, underscoring the role of cogging force compensation in enhancing tracking accuracy for iron-core linear motors.

Figure 13 and Figure 14 display the control inputs and disturbance estimates of each controller, respectively. Notably, C2 and C4, which incorporate system model compensation and online parameter estimation, exhibit lower disturbance estimates than C1. Among them, with a control input amplitude approximately consistent across controllers, C4—further benefiting from cogging force compensation—achieves the smallest disturbance estimate.

The tracking performance metrics during 5–50 s and 40–50 s ($e_{m5}$, $e_{r5}$, $e_{m40}$, $e_{r40}$) are summarized in Table 4. The results indicate that, although C3 demonstrated excellent performance in trajectory tracking, its requirement for smaller initial gains during the positioning phase resulted in larger position tracking errors. In contrast, C1, C2, and C4 demonstrated progressively better trajectory tracking performance as the system model was enriched and parameter adaptation was introduced. The Zeta-backstepping-based C4 and the traditional backstepping-based C3 showed comparable steady-state performance, indicating that the proposed control scheme not only achieves the tracking performance of traditional backstepping controllers but also excels in scenarios involving mixed positioning and tracking tasks. Furthermore, by refining the system model and incorporating parameter adaptation, the proposed scheme effectively enhances the system’s position tracking performance.

#### 4.3.3. Case 3: Disturbance Rejection

This case is designed to test the disturbance rejection capability of the proposed control scheme during operation, thereby validating its robustness. The same tracking trajectory as in Case 2, $x_{d}\left(t\right)=0.02\left[2-\cos\left(\pi t\right)\right]$ (m), is used here. However, during the stable operation phase, a disturbance torque is introduced by injecting $d_{s}\left(t\right)=0.15\sin\left(2\pi \left(t-30\right)\right)$ for 30≤t≤40 into the control input *u*. This comparison is again conducted under approximate critical damping conditions, with parameter settings consistent with Case 2.

**Figure 15:**
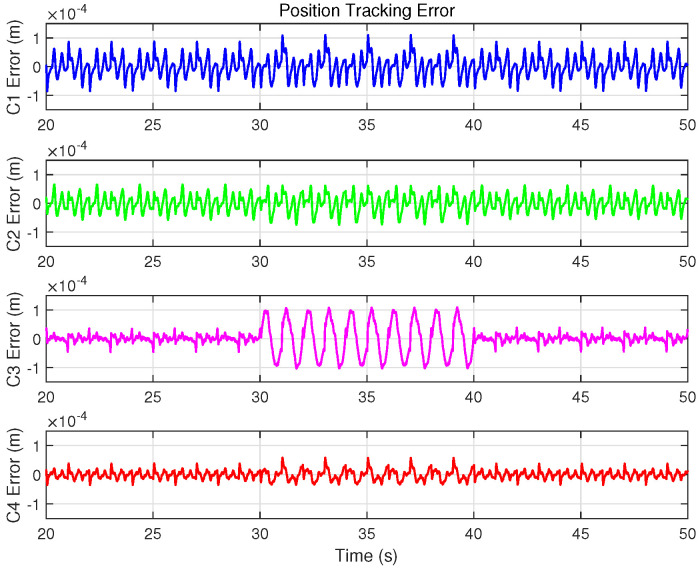
Position tracking errors of four controllers under the sinusoidal trajectory with disturbance addition during trajectory tracking phase in Case 3.

**Figure 16:**
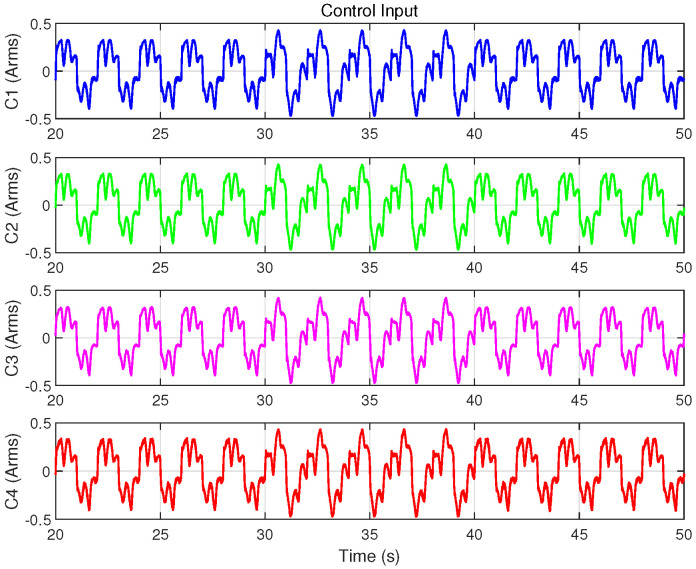
Control input of the four controllers in Case 3.

**Figure 17:**
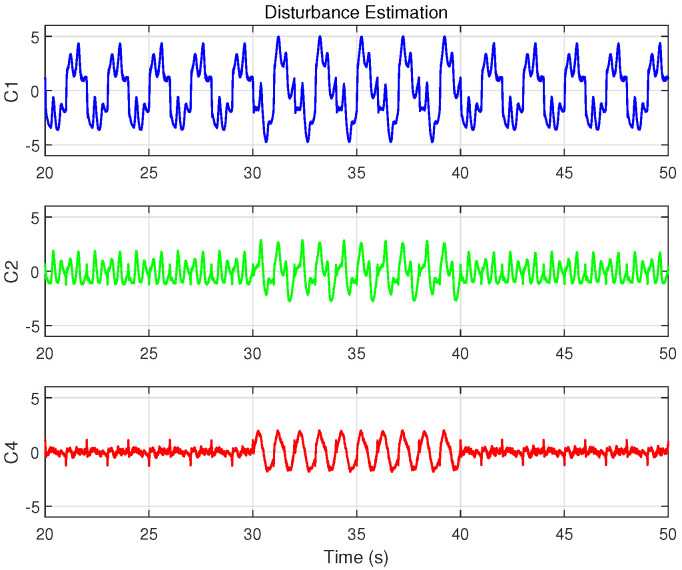
Disturbance estimation results of the controllers with ISMO in Case 3.

In addition to programming the desired trajectory $x_{d}\left(t\right)$ and control algorithms in TwinCAT, this case requires recording the program execution time and injecting the disturbance torque $d_{s}\left(t\right)$ into the control input *u* within the specified time period. Experimental data are recorded using the TwinCAT software, and the corresponding results are shown in Figure 15, Figure 16 and Figure 17 and Table 5.

As depicted in Figure 13, the disturbance introduced, with an amplitude of 0.15 A, is significant compared to the approximately 0.4 A control input during sinusoidal trajectory tracking, effectively simulating intense external disturbances. Figure 15 illustrates the position tracking error responses of the four controllers before and after the disturbance was introduced. The results indicate that, upon disturbance injection, the tracking error of the IARC controller C3, which does not employ ISMO, increased significantly, whereas the tracking errors of C1, C2, and C4 showed mild variations, ensuring stable and continuous system operation. Notably, the tracking error variation of model-compensated controllers C2 and C4 was smaller than that of C1, with C4 exhibiting the least deviation, highlighting the advantages of cogging force compensation. Figure 16 and Figure 17 display the control inputs and observer estimation results of the corresponding controllers, respectively. It is evident that the ISMO in controllers C1, C2, and C4 quickly detected and compensated for the disturbance, maintaining tracking performance. In contrast, C3, lacking specific disturbance compensation, exhibited significant error fluctuations.

Tracking performance metrics during 20–50 s, 30–40 s and 40–50 s ($e_{m20}$, $e_{r20}$, $e_{r30}$, $e_{m40}$, $e_{r40}$) are summarized in Table 5. The data show that C3 demonstrated excellent tracking performance in the absence of disturbances, even exhibiting the lowest RMS value after the disturbance was removed (i.e., $e_{r40}$). However, during the disturbance period, C3’s tracking performance was the worst, even inferior to the non-model-based C1. This underscores the exceptional disturbance suppression capability of the designed ISMO. In summary, this experiment validates that the proposed control scheme not only ensures trajectory tracking performance but also significantly enhances the system’s disturbance rejection ability, thereby improving overall robustness.

Through the above experiments, it can be seen that the proposed control strategy provides an effective solution for point-to-point positioning and trajectory tracking control in practical applications of direct-drive systems. For example, in surface mount technology (SMT), where linear motors drive placement heads for component installation, this control approach ensures controlled overshoot during the positioning process, enabling precise point-to-point motion and accurate placement of components, thereby improving product yield and production efficiency. Similarly, in computer numerical control (CNC) machining, linear motors drive tools or workpieces for high-speed, high-precision processing. Through precise point-to-point positioning and robust trajectory tracking control, the system can maintain disturbance resistance, enhancing both processing accuracy and speed.

## 5. Conclusions

Direct-drive servo systems, increasingly employed in biomimetic systems such as robotic applications, face challenges from nonlinearities, parameter uncertainties, and unknown disturbances. To address these challenges, this study proposes an adaptive disturbance rejection Zeta-backstepping control scheme. Using an iron-core permanent magnet linear synchronous motor (PMLSM) as the demonstration system, a detailed dynamic model of the PMLSM is developed, incorporating compensation for friction and cogging forces.

To address parameter uncertainties, an indirect adaptive strategy is introduced, employing a recursive least squares algorithm with a forgetting factor to ensure reliable parameter convergence while decoupling controller design from parameter estimation. Additionally, an integral sliding mode observer (ISMO) is designed to estimate and compensate for external disturbances and other uncertainties without assuming differentiability. This observer achieves finite-time convergence for state estimation and enhances disturbance rejection. A Zeta-backstepping approach is then adopted for controller synthesis, ensuring tracking error stability while enabling explicit overshoot control through an adjustable damping ratio, following a predefined parameter selection framework. Comprehensive experiments conducted on a real PMLSM platform validate the effectiveness of the proposed scheme, demonstrating controlled overshoot, improved tracking accuracy, and enhanced disturbance rejection. Practical applications are further illustrated using SMT and CNC systems as examples, showcasing the strategy’s applicability in point-to-point positioning and trajectory tracking tasks.

In conclusion, this study systematically addresses nonlinearity and disturbance challenges while achieving parameterized damping ratio dynamics, providing theoretical insights and practical guidance for reliable, high-precision control in direct-drive systems and biomimetic applications. Future work will focus on further refining the method for enhanced optimization and broader applicability, contributing to the continued advancement of motion control in direct-drive and biomimetic systems.

## Figures and Tables

**Table 1:** Detailed information of devices within the experimental platform.

Devices	Model	Manufacturer	City	Country
Computer’s CPU	Ryzen 5 1600	Advanced Micro Devices, Inc.	Santa Clara (CA)	United States
Linear motor	SGLFW-50A200B	Yaskawa Electric Co., Ltd.	Shanghai	China
Servo drive	G-OBO13/230FEHN1	Elmo Motion Control Ltd.	Petah Tikva	Israel
Linear scale	RGS20	Renishaw plc	Shanghai	China
Readhead	RGH22B	Renishaw plc	Shanghai	China

**Table 2:** Identified model parameters of cogging force.

Parameters	k=1	k=2	k=3	k=4	k=5	k=6	k=7
$A_{rk}\left(N\right)$	1.806	7.612	2.418	0.813	1.303	1.255	1.148
$\omega_{k}$	42.50	44.74	46.98	51.45	53.69	89.48	98.43
$\varphi_{k}$	1.671	4.424	3.695	−1.374	0.204	−1.509	−0.346

**Table 3:** System model parameters setting.

	$\theta_{1}$	$\theta_{2}$	$\theta_{3}$	$\theta_{4,1}$	$\theta_{4,2}$	$\theta_{4,3}$	$\theta_{4,4}$	$\theta_{4,5}$	$\theta_{4,6}$	$\theta_{4,7}$
$\theta_{0}$	12	15	2	0.1	1.2	0.4	0.2	0.2	0.2	0.2
$\theta_{\min}$	5	10	0.1	−2	−3	−2	−2	−2	−2	−2
$\theta_{\max}$	20	30	5	2	3	2	2	2	2	2

**Table 4:** Quantitative evaluation indexes of Case 2.

Controller	$e_{m5}$ (μm)	$e_{r5}$ (μm)	$e_{m40}$ (μm)	$e_{r40}$ (μm)
C1_ISMO_NoModel	81.10	35.50	81.10	35.45
C2_ISMO_NoCogging	72.01	27.10	61.08	24.48
C3_IARC	215.74	11.62	47.66	11.25
C4_Proposed	41.10	12.40	36.38	11.13

**Table 5:** Quantitative evaluation indexes of Case 3.

Controller	$e_{m20}$ (μm)	$e_{r20}$ (μm)	$e_{r30}$ (μm)	$e_{m40}$ (μm)	$e_{r40}$ (μm)
C1_ISMO_NoModel	110.41	35.83	39.57	87.06	33.74
C2_ISMO_NoCogging	74.82	28.49	32.89	62.97	25.34
C3_IARC	109.42	40.67	68.67	44.85	11.39
C4_Proposed	58.83	14.67	19.35	38.29	11.50

## Data Availability

Data are contained within the article.

## Appendix A. Proof of Theorem 1

**Proof.** The proof for Theorem 1 proceeds in three steps: Step 1: Convergence of the integral sliding mode variable s(t) Define the Lyapunov function for the sliding mode variable s(t) as $V_{s}\left(t\right)=\frac{1}{2}s^{2}\left(t\right)$. According to (20), the time derivative of s(t) is

(A1) $$\dot{s}\left(t\right)={\dot{\xi }}_{1}\left(t\right)+\eta_{o}\mathrm{sign}\left(\xi_{1}\left(t\right)\right)+\iota_{1}\xi_{1}\left(t\right).$$

Combining with (18) and (21), we obtain the derivative of $V_{s}\left(t\right)$ is

(A2) $${\dot{V}}_{s}=s\dot{s}=s\left(\xi_{2}-\left(\varrho_{o}+\left|{\hat{x}}_{2}\right|\right)\mathrm{sign}\left(s\right)\right)\leq \left|s|\right|\xi_{2}|-\left(\varrho_{o}+\left|{\hat{x}}_{2}\right|\right)\left|s\right|.$$

Since $|\xi_{2}\left|=\right|{\hat{x}}_{2}-x_{2}\left|\leq \right|{\hat{x}}_{2}\left|+\right|x_{2}|$, it follows that

(A3) $${\dot{V}}_{s}\leq -\left(\varrho_{o}-\left|x_{2}\right|\right)\sqrt{2V_{s}}\leq -\varrho \sqrt{2V_{s}}$$

where $\varrho =\varrho_{o}-\sqrt{E_{k}/M_{\min}}>0$. Noting the definition of s(t) in (20) reveals that s(0)=0. Thus, based on (A3), we can deduce that

(A4) $$s\left(t\right)=0,\;\dot{s}\left(t\right)=0,\;\forall t\geq 0.$$

Therefore, the sliding variable remains on the sliding manifold from the initial moment onward, effectively eliminating the reaching phase and ensuring invariance with respect to the lumped uncertainty $d_{l}$ for t>0. Step 2: Finite-time convergence of estimation error $\xi_{1}$ From (A1) and (A4), we have:

(A5) $${\dot{\xi }}_{1}\left(t\right)=-\eta_{o}\mathrm{sign}\left(\xi_{1}\left(t\right)\right)-\iota_{1}\xi_{1}\left(t\right).$$

Define the Lyapunov function for $\xi_{1}$ as $V_{o1}\left(t\right)=\frac{1}{2}\xi_{1}^{2}\left(t\right)$. Differentiating $V_{o1}\left(t\right)$ with respect to time yields:

(A6) $${\dot{V}}_{o1}=\xi_{1}\left(-\eta_{o}\mathrm{sign}\left(\xi_{1}\right)-\iota_{1}\xi_{1}\right)=-\eta_{o}\left|\xi_{1}\right|-\iota_{1}\xi_{1}^{2}<-\eta_{o}\sqrt{2V_{1}}$$

which implies that $V_{o1}\left(t\right)=0$ after a finite time $t_{1}$ defined as follows.

(A7) $$t_{1}<\frac{1}{\eta_{o}}\left|\xi_{1}\left(0\right)\right|$$

Thus, we conclude that $\xi_{1}\left(t\right)$ converges to zero in finite time $t_{1}$, after which ${\dot{\xi }}_{1}=0$, satisfying (24). Step 3: Finite-time convergence of estimation error $\xi_{2}$ By the result of step 2 and combining (21) and (24), it can be obtained that $\omega_{1}=-\xi_{2},\;t\geq t_{1}$. Define the Lyapunov function for $\xi_{2}\left(t\right)$ as $V_{o2}\left(t\right)=\frac{1}{2}\xi_{2}^{2}\left(t\right)$. Taking the derivative and applying (19) and (22), one has

(A8) $${\dot{V}}_{o2}=\xi_{2}\left(\iota_{2}\omega_{1}+\omega_{2}-d_{l}\right)\;=\;\xi_{2}\left(-\iota_{2}\xi_{2}-\kappa_{o}\mathrm{sign}\left(\xi_{2}\right)-d_{l}\right)\;\leq \;-\left(\kappa_{o}-\delta_{h}\right)\left|\xi_{2}\right|\leq -\kappa \sqrt{2V_{2}}$$

where $\kappa =\kappa_{o}-\delta_{h}>0$. Similar to step 2, we can deduce that $V_{o2}\left(t\right)=0$ for $t>t_{2}$, where $t_{2}<t_{1}+\frac{1}{\kappa }\left|\xi_{2}\left(t_{1}\right)\right|$. Consequently, it follows that $\xi_{2}\left(t\right)$ converges to zero in finite time $t_{2}$, after which ${\dot{\xi }}_{2}=0$, satisfying (25). Finally, from (22), we see that $d_{l}=\iota_{2}\omega_{1}+\omega_{2},\;t>t_{2}$, i.e., (26) holds. □

Thus, the proof is complete, demonstrating that the constructed observer (17) achieves finite-time convergence of the estimation errors and provides accurate state estimation for the system.
